# Stable Compressible Liquids Made of Hierarchical MOF Nanocrystals

**DOI:** 10.1021/acsami.4c21181

**Published:** 2025-04-22

**Authors:** Heting Xiao, Xi-feng Liang, Wei Zhou, Hebin Jiang, Daniel S. Parsons, Haixia Yin, Bitao Lu, Yueting Sun

**Affiliations:** a School of Traffic & Transportation Engineering, Central South University, Changsha, Hunan 410083, China; b School of Engineering, 1724 University of Birmingham, Edgbaston, Birmingham, West Midlands B15 2TT, United Kingdom

**Keywords:** compressible liquids, metal−organic frameworks, water intrusion, water stability, colloidal stability, hierarchical structures, energy absorption, porous liquids

## Abstract

Compressible liquids can be produced by dispersing nanoparticles containing hydrophobic pores as colloidal suspensions in water. Due to the water intrusion into the hydrophobic nanopores under pressure, these compressible liquids exhibit significantly greater compressibility than traditional liquids, lending them to energy storage and absorption applications. Metal–organic frameworks (MOFs) such as ZIF-8 have been proposed for this application due to their large porosity, but their physical and chemical stability in aqueous environments presents challenges, prone to hydrolysis or separation from the liquid phase. In this work, the stability concerns of ZIF-8 used for compressible liquids have been circumvented by producing nanoparticles of mesoporous ZIF-8 by a template-directed synthesis. The stability, compressibility, and intrusion kinetics were compared between ZIF-8 with and without mesopores. The mesoporous ZIF-8, uniquely containing hydrophobic micropores and hydrophilic mesopores, presents compressibility comparable to that of conventional ZIF-8 due to the hydrophobic micropores but has the added benefit of significantly increased physical and chemical stability due to the hydrophilic mesopores. The presence of mesopores slightly reduces the water intrusion pressure and accelerates the kinetics that can benefit the cyclic compressibility for vibrations or repeated impact applications as water molecules reversibly intrude and extrude the micropores. This work can inspire future endeavors on understanding and developing compressible and porous liquids with sufficient stability for practical uses.

## Graphical abstract

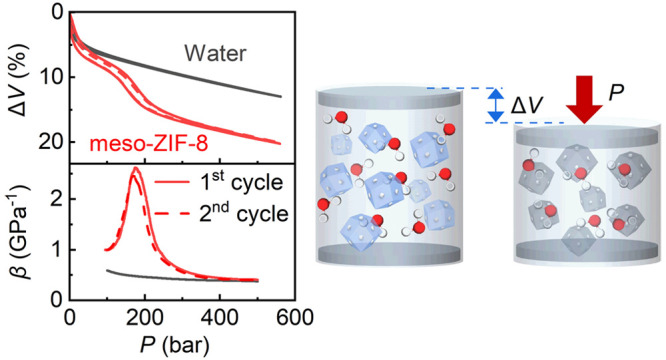

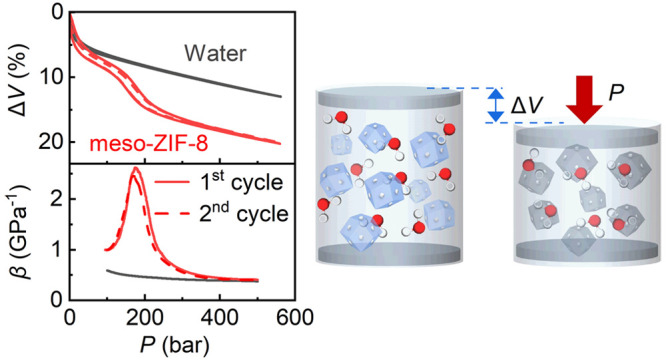

## 1 Introduction

Water is almost incompressible due to the very little empty space between closely packed water molecules. However, strategies of introducing permanent porosity into water^1,2^ will make it possible to produce highly compressible water. Porous liquids, initially proposed by the James group,^3^ demonstrated that permanent microporosity can be extended from solid-state materials to the liquid phase.^4,5^ Current approaches to preserving permanent microporosity in porous liquids are mostly sterics-based, using microporous nanocrystals or organic cage molecules dispersed in bulky solvents such as organic solvents or ionic liquids that do not enter the micropores.^6–8^ This approach is, however, not transferable to water due to its small molecule size. But if the micropores are hydrophobic, it will be favorable for water to stay in the bulk instead of occupying the pores. This thermodynamic approach will allow water to gain permanent porosity,^2^ and importantly, such porosity can be consumed by water molecules once sufficient external pressure is applied, thus giving a high compressibility to water due to its substantial volume reduction during the pressurized intrusion of water molecules into the hydrophobic micropores.^9,10^

Such highly compressible liquid can be obtained by dispersing hydrophobic porous solids in water,^1^ as a type 3 porous liquid.^3,4^ To form a stable suspension, the porous solids should be nanosized and hydrophilic surfaces are usually preferable. Metal–organic frameworks (MOFs) can be a good candidate, which can provide hydrophobic micropores as well as tunable crystal size and surface properties. MOFs are composed of metal ions and organic ligands, forming frameworks with high pore volumes and high specific surface areas, with applications in gas storage and separation,^11^ liquid separation,^12^ drug delivery,^13^ catalysis,^14^ etc. The pressurized intrusion of liquid water in hydrophobic MOFs has been studied in the past decade for energy storage,^15,16^ shock absorption,^17,18^ and electrification,^19^ etc. The intrusion behavior can be tuned by adding salts and alcohols to the aqueous solution in which the MOF is immersed.^20–22^ Zeolitic imidazolate frameworks (ZIFs), a class of MOFs comprising metal ions and imidazolate linkers that form framework structures analogous to zeolitic topologies, are the main materials adopted for water intrusion so far.^16,23^ ZIF-8 is the first MOF studied under water intrusion,^16^ which is composed of zinc ions (Zn^2+^) and 2-methylimidazolate (mIm^–^) linkers. However, one of the inherent challenges lies in the water stability of MOFs, as the bonds between their metal ions and organic linkers are prone to hydrolysis in aqueous solution.^24^ Although ZIFs are known for their excellent stability, they can still degrade over the long-term or under harsh conditions. For example, the water stability of ZIF-8 is influenced by the ZIF:H_2_O ratio, with a higher ratio resulting in a slower degradation, and the majority of degradation occurs within the initial hour of water immersion, after which the degradation slows down.^25–28^

A compressible liquid of MOFs immersed in water requires the MOF nanoparticles to be physically and chemically stable. Colloidal stability is generally contributed by electrostatic repulsion, steric hindrance between surface species, and favorable interactions with solvent, etc.^29^ Existing works have demonstrated approaches to improving colloidal stability by controlling particle size,^30,31^ tuning the liquid phase including those that can penetrate MOF pores,^29^ and modifying the solid–liquid interface by polymer or lipid coating.^2,32,33^ Preventing MOFs from chemically degrading in water is also an active area of research.^34,35^ Many postsynthetic processes have been reported using hydrophobic groups, such as functionalizing DUT-4 with hydrophobic organosilicon moieties,^37^ ZIF-8 with alkyl chains,^38^ and replacing the 2-methylimidazolate on ZIF-8 crystal surface with the hydrophobic 5,6-dimethylbenzimidazolate.^39^

In this paper, we propose a new approach to improving the physical and chemical stabilities of hydrophobic MOFs in liquid water, that is, by introducing hydrophilic mesoporosity into MOF nanocrystals, ultimately yielding a highly stable compressible aqueous solution. Different from traditional stabilization approaches that rely on exterior surface coating, our strategy is to create new “interior” surfaces that have strongly favorable solvent interaction so that colloidal stability can be improved without turning the crystal into a “composite” or controlling the electrostatic repulsion from surface charges or the steric repulsion from surface groups. Therefore, in contrast to previous studies on the water intrusion of a hierarchical zeolite where the additional mesoporosity is hydrophobic,^40–42^ we chose to introduce highly hydrophilic mesopores into MOF nanocrystals, where water as the solvent can access and interact with the interior of the mesopores. Such a design essentially allows water to “dissolve” the nanocrystal, resembling the solubility of large macromolecules based on exothermic interactions. We also envision that the highly favorable hydrogen bonds between the new surface and the solvating water molecules may also contribute to the resistance to hydrolysis, akin to the role of hydrophilic coating reported before on the improved stability of ZIF-8 in water.^43^

To test the envisioned approach, we synthesized hierarchical ZIF-8 as a proof of concept, which has hydrophobic micropores and hydrophilic mesopores, as well as a small nanocrystalline size to ensure a good foundation for dispersion stability. MOFs containing hierarchical defects, including introducing mesopores into microporous crystals,^44–46^ can be produced with *de novo* synthetic strategies either with template^47^ or template-free^48^ and with postsynthetic strategies such as chemical etching,^49^ selective^50^ or nonselective^51^ hydrolysis, thermal annealing,^52^ and stepwise ligand exchange,^53^ etc. We adopted the surfactant soft template method under aqueous conditions, eliminating the need for prefabricated template materials. By studying the compressible liquid consisting of such mesoporous ZIF-8 crystals in water, we successfully demonstrated its improved physical and chemical stabilities, long-term compressibility, and, interestingly, the different kinetics of water intrusion in hierarchical structures, where the presence of mesopores accelerates the water transport, beneficial to the energy absorption applications of such compressible liquids under highly dynamic and cyclic loading conditions. It is envisaged that the proposed approach can be applied more widely to other MOFs that exhibit solvent intrusion and lead to various compressible liquids with different characteristics. To our knowledge, this is the first time that MOF stability, including colloidal and chemical stability, has been investigated in the context of compressible liquids, addressing an outstanding research gap that is critical to applications. Notably, this work is relevant to a number of fields, including liquid compressibility in the field of water intrusion,^1,18^ MOF hydrolysis in the field of porous materials,^24,34,35^, and colloidal stability in the field of porous liquids^2,31^ and nanofluids,^54^ and the synthesis and applications of hierarchical MOFs^44–46^ where their surface properties^55–57^ can be critical.

## 2 Experiment Section

### 2.1 Materials

Zinc nitrate hexahydrate (Zn (NO_3_)_2_·6H_2_O, 99%), hexadecyltrimethyl­ammonium bromide (CTAB, 99%), triethylamine (TEA, 99%), 2-methylimidazole (HmIm, 98%), l-histidine (L-his, 99%), ethanol (EtOH, 99.7%), methanol (MeOH, 99.5%), and ultrapure water were purchased from Shanghai Macklin Biochemical Co., Ltd. All solvents and chemicals are of reagent quality and were used without further purification. Deionized water was used to prepare the compressible liquid throughout this work.

### 2.2 ZIF-8 Synthesis

ZIF-8 was synthesized following a procedure adapted from a previous study.^58^ A solution of zinc nitrate hexahydrate (3.0 g, 10 mmol) in methanol (200 mL, 5 mol) was rapidly added to a solution of 2-methylimidazole (6.6 g, 80 mmol) in methanol (200 mL, 5 mol). The mixture was stirred for 1 h at 55 °C. The precipitate was recovered by centrifugation (10 000 rpm, 10 min), washed three times with methanol, and dried overnight in air. The obtained product was activated by heating at 150 °C under vacuum for 24 h.

### 2.3 Mesoporous ZIF-8 Synthesis

The synthetic method was modified from Adnan et al.^59^ in which zinc nitrate hexahydrate (1.48 g, 4.97 mmol), CTAB (1 g, 2.74 mmol), and l-histidine (1.688 g, 10.88 mmol) were dissolved in deionized water (100 mL) with stirring. A second solution was prepared by dissolving 2-methylimidazole (3.24 g, 39.46 mmol) and triethylamine (4.00 g, 39.53 mmol) in deionized water (100 mL) with stirring. The two solutions were mixed with vigorous stirring for 12 h at ambient temperature. The white precipitate was recovered by centrifugation (10 000 rpm, 10 min) and then washed with an ethanol–water solution 50% (v/v) at 60 °C for 3 h. The obtained product was activated by heating at 150 °C under vacuum for 24 h.

### 2.4 Materials Characterization

The mesopores were characterized using field-emission transmission electron microscopy (FE-TEM) on a FEI Tecnai G2F20 microscope (FEI, Hillsboro, OR, USA) with an accelerating voltage of 200 kV. Particle morphology and size distributions were analyzed by scanning electron microscopy (SEM) on a TESCAN Mira3 XMH microscope (TESCAN, Brno, Czechia) equipped with an energy-dispersive X-ray (EDS) detector. Porosimetry measurements were made on a BSD-600 instrument (BSD Instruments, China) with nitrogen at 77 K. The surface area was obtained by the Brunauer–Emmett–Teller (BET) method, and the pore size distribution was obtained by the Barrett–Joyner–Halenda (BJH) model. The micropore volume is calculated using T-plot theory, and the mesopore volume is calculated using BJH theory. Powder X-ray diffraction (PXRD) patterns were characterized using a PANalytical Empyrean X-ray diffractometer equipped with a Cu Kα source at a scan rate of 10° per minute using a 2θ step-size of 0.01–0.03° in the range 2θ = 5–30°. Fourier-transform infrared (FTIR) spectroscopy was performed on a Bruker Alpha-P ATR FTIR spectrometer in the range 400–4000 cm^–1^. ζ potentials were determined by Brookhaven NanoBrook Omni instrument at ambient temperature with the concentration of 0.1 wt % ZIFs in deionized water.

### 2.5 Compression Tests

Compression tests were conducted in a custom pressure chamber with two pistons using an MTS 858 testing machine. A cylindrical sample (6 mm diameter, 3 mm length) comprising 25 mg of ZIF-8 powder was used (Figure S1). The pressure on the sample increased from ambient to 600 bar by pressing the piston at a constant speed 0.5–2500 mm min^–1^ (corresponding to a strain rate of 3 × 10^–3^–14 s^–1^), which then moved back at the same speed to reduce the pressure to ambient. The pressure and displacement were recorded for the compression–decompression cycle to plot the volume change as a function of pressure (*P*–Δ*V* curves) for the compressible liquid.

## 3 Results and Discussion

### 3.1 Meso-ZIF-8 Characterization

Mesoporous ZIF-8, herein denoted as meso-ZIF-8, was synthesized in an aqueous solution by a templating method employing the surfactant CTAB. CTAB serves as a structure-directing agent by forming micelles, where the hydrophobic cetyl chains aggregate to form the core of the micelle, while the hydrophilic trimethylammonium groups form the outer shell, as illustrated in Figure 1a. l-Histidine is also added as a chelating agent, facilitating stable interactions between the surfactant micelles and ZIF precursors for a synergistic template effect. The template is removed postsynthesis by washing with an ethanol–water mixture, yielding meso-ZIF-8.

**Figure 1:**
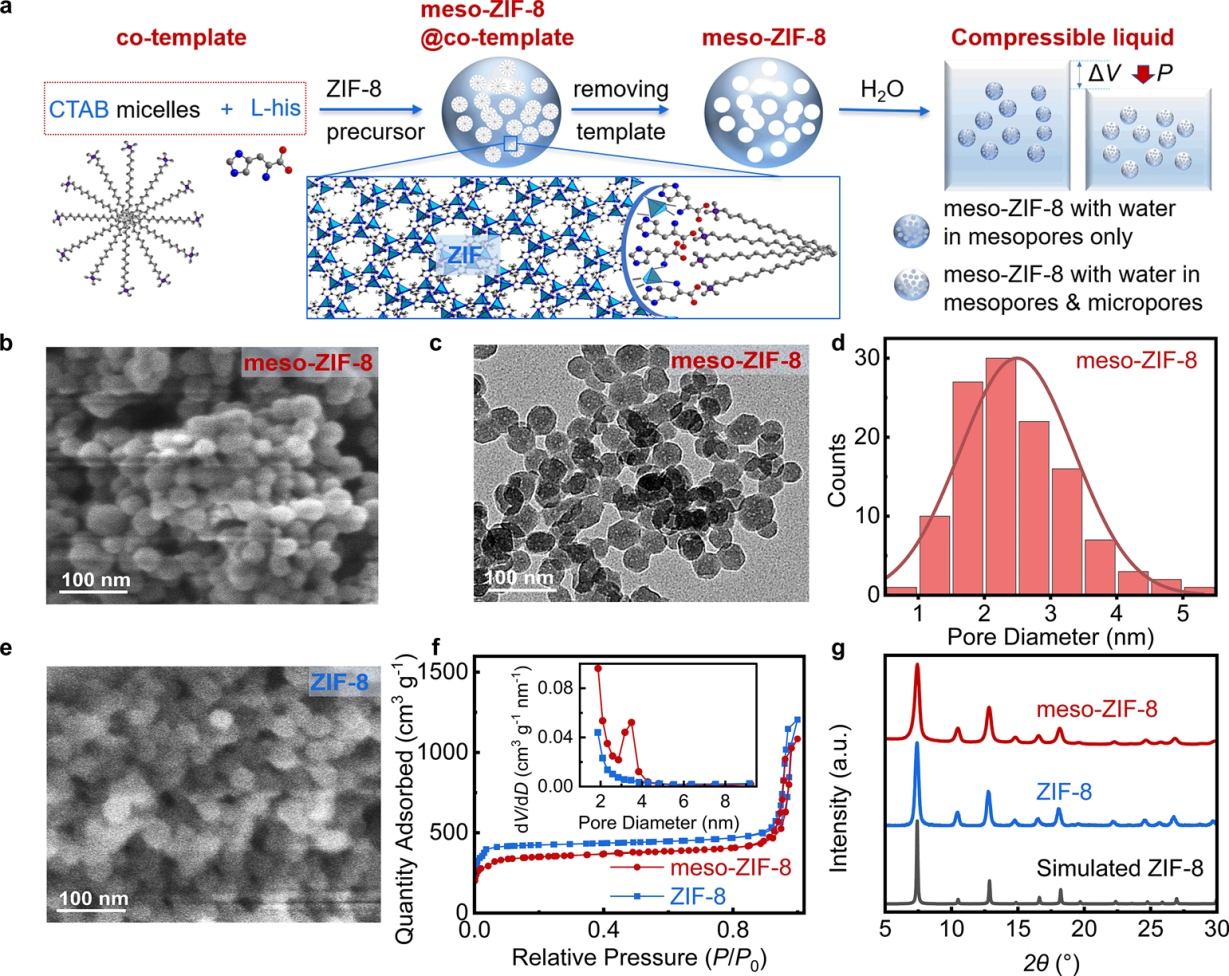
Synthesis method and material characterization of meso-ZIF-8 and ZIF-8 nanocrystals. (a) Schematic showing the synthesis of meso-ZIF-8 by a templating method. (b) SEM image of meso-ZIF-8, with particle size distribution shown in Figure S2a,c. (c) TEM image of meso-ZIF-8. (d) Mesopore diameter distribution of meso-ZIF-8 calculated from TEM (Figure S2e,f), (e) SEM image of ZIF-8, with particle size distribution shown in Figure S2b,d. (f) Nitrogen adsorption–desorption isotherms of meso-ZIF-8 and ZIF-8, with the inset showing pore diameter distribution. (g) Normalized PXRD patterns of meso-ZIF-8 and ZIF-8.

The meso-ZIF-8 synthesized by this method possesses a nanocrystalline size similar to ZIF-8 produced by the conventional synthesis (Figure 1b,e), with particle size as small as 32 nm (Figure S2). The mesopore diameters in the particles are 2.3 nm as determined by TEM (Figures 1c, Figure S2) and 3.6 nm by N_2_ adsorption (Figure 1f). PXRD confirms that meso-ZIF-8 adopts a ZIF-8 crystal structure (Figure 1g).

The presence of mesopores in meso-ZIF-8 leads to a reduction in the specific surface area *S*_BET_ to 1359 m^2^ g^–1^ compared with conventional ZIF-8 (1746 m^2^ g^–1^). The micropore volume *V*_0.35–2 nm_ for meso-ZIF-8 (0.47 cm^3^ g^–1^) is also lower than that for conventional ZIF-8 (0.61 cm^3^ g^–1^), after gaining 0.12 cm^3^ g^–1^ of mesopores, as illustrated in Figure 1f. The full width at half-maximum of Bragg peaks in the meso-ZIF-8 PXRD patterns is greater than in conventional ZIF-8 PXRD patterns (Figure 1g), indicating a reduction in the size of crystal domains in the particles, in line with what would be expected upon introducing mesopore defects in the material.

### 3.2 Improved Stability

Conventional ZIF-8 exhibits a high water contact angle (∼133°), as previously reported,^1^ and further demonstrated in Figure 2a, whereas meso-ZIF-8 has a much lower water contact angle (∼71°) as shown in Figure 2b. The difference in contact angle is indicative that introducing mesopores into ZIF-8 makes the material more hydrophilic, as we aimed for. The more hydrophilic behavior of meso-ZIF-8 is likely related to defects lining the mesopores, which would chiefly constitute hydrophilic hydroxide moieties coordinating with zinc ions in the framework. This hypothesis is supported by the negative ζ potential value of meso-ZIF-8 and its enhanced O–H signal in FTIR, which are not seen in the conventional ZIF-8 (Figure S3). As identified previously,^1,60^ the terminal zinc ions on the ZIF-8 surface are coordinated to either hydroxyl or carbonate groups. In the absence of imidazole linkers, a surface rich in terminal zinc-hydroxide groups can be created, resulting in a more hydrophilic ZIF-8 surface. However, it is also worth noting a most recent simulation which reveals the intrinsic role of mesoporosity on the hydrophilicity of porous particles,^56^ which may have also contributed to the observed hydrophilicity of our meso-ZIF-8 sample.

**Figure 2:**
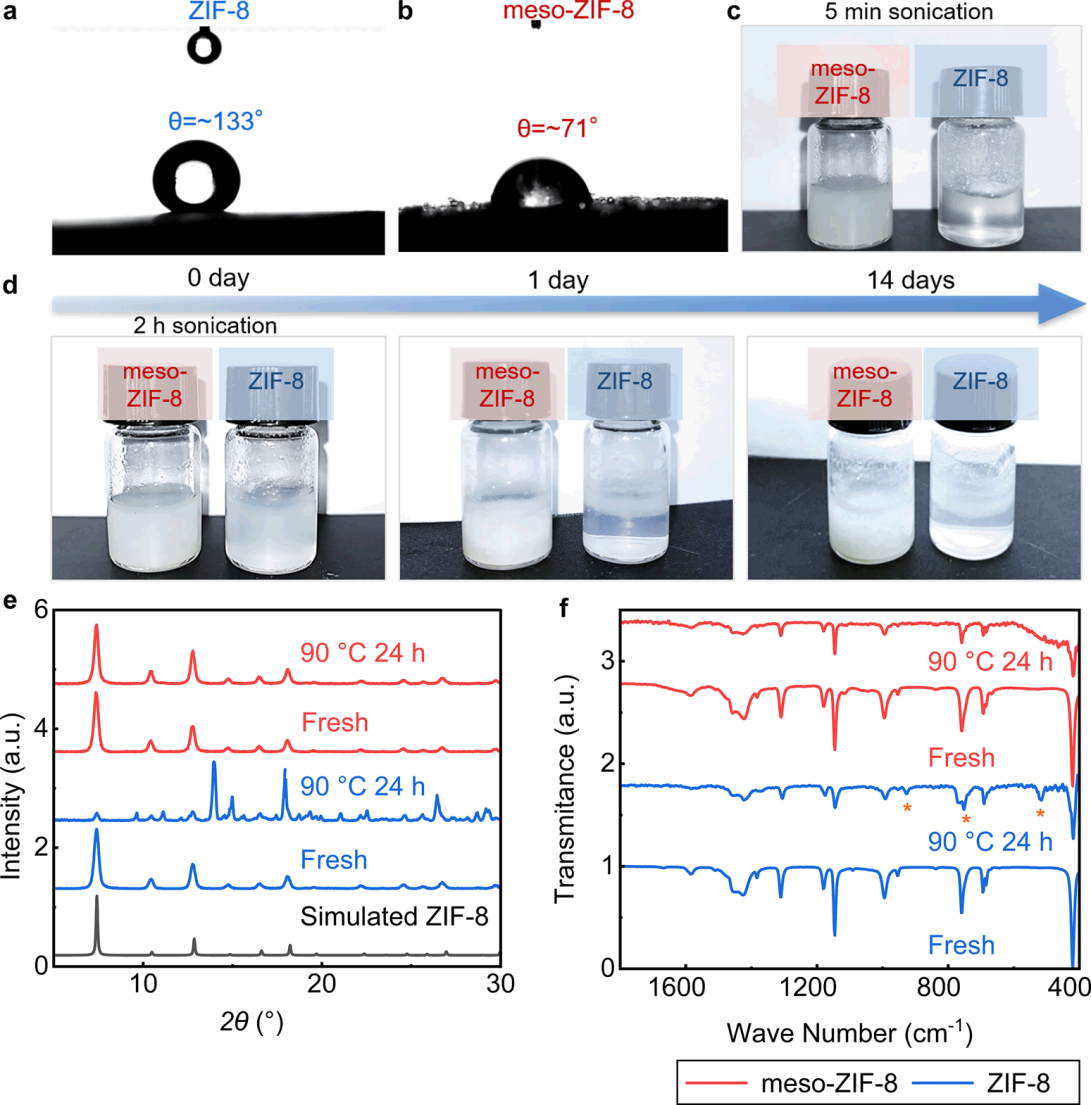
Physical and chemical stabilities of meso-ZIF-8 (red) and ZIF-8 (blue) in liquid water: (a, b) water contact angle measurements, (c) photos after 5 min of sonication and (d) 2 h of sonication at a concentration of 1.6 wt %, with additional photos after 1 day and 14 days, whose chemical stability has been checked by PXRD in Figure S5a, (e) PXRD patterns and (f) FT-IR after 24 h at 90 °C in liquid water at a concentration of 29.7 wt %.

As expected, the highly hydrophilic mesopore surface permits meso-ZIF-8 to form a stable colloidal suspension in liquid water. Sonication of meso-ZIF-8 in water for 5 min leads to a uniform suspension (Figure 2c), whereas conventional ZIF-8 must be sonicated for 2 h to form a uniform suspension (Figure 2d). The stability of the suspension is also greater for meso-ZIF-8, which remains a stable, uniform suspension for 14 days, whereas conventional ZIF-8 will separate from suspensions after just 1 day (Figure 2d). The meso-ZIF-8 did not form a permanently stable suspension though (Figure S4), which is understandable because as a multiphase system, suspension is inherently unstable in the thermodynamic sense due to the raised free energy relative to that of the seperate phases,^61^ so its stability needs to be described at a time frame. Fundamentally, the stability of suspension is related to a variety of factors,^61,62^ including (i) wetting associated with the attraction force between the solid and the liquid, (ii) dispersion associated with the electrostatic repulsion between particles, (iii) density matching between the solid and the liquid that prevents sedimentation, (iv) particle size which is proportional to the sedimentation velocity according to Stokes’ law,^63^ and (v) the rheology of the liquid. In this work, ZIF-8 has a density similar to that of liquid water and a small crystal size, providing a good starting point, and the additional hydrophilic mesopores make the difference. We visually examined the dispersion stability in our experiments, but quantitative measurements can also be adopted such as PXRD and near-infrared absorbance under centrifugation.^31,62^

Tests were performed at elevated temperatures to probe the chemical stability of meso-ZIF-8 and conventional ZIF-8 in aqueous colloidal suspensions. The presence of mesopores in meso-ZIF-8 leads to enhanced chemical stability compared with conventional ZIF-8, owing to the presence of hydroxide capping, giving rise to thermodynamically favorable hydrogen-bonding interactions between the ZIF-8 surface and the solvating water molecules. Fundamentally, this mechanism resembles traditional postsynthetic surface functionalization where hydrophilic polymer coating containing hydroxyl groups stabilizes ZIF-8 in water.^43,64^ As demonstrated by PXRD performed on the recovered solids in Figure 2e, heating the colloidal suspension at 90 °C for 24 h leads to the destruction of conventional ZIF-8, whereas meso-ZIF-8 retains its crystal structure, indicating an enhanced resistance to hydrolysis. The PXRD pattern recorded on conventional ZIF-8 following hydrolysis shows that new phases have been produced from the destruction of the material. FTIR spectra further illustrate the difference in chemical stability between meso-ZIF-8 and conventional ZIF-8. The FTIR spectra for meso-ZIF-8 are analogous before and after heating with retention of all of the dominant bands; in contrast, the FTIR spectrum recorded on ZIF-8 following heating shows several new bands, as marked with an asterisk in Figure 2f. Difference in chemical stability at room temperature has also been detected after 14 days using the samples in Figure 2d (Figure S5a).

Ultimately, the introduction of hydrophilic mesopores into ZIF-8 improves resistance to hydrolysis and enhances the stability of colloidal suspensions of the ZIF particles in aqueous solution. The improvement in stability provides excellent potential for using these colloidal systems in energy absorption and storage applications.^1,17^

### 3.3 Liquid Compressibility

Since ZIF-8 has hydrophobic micropores, water does not enter its pores at ambient pressure, but applying sufficient pressure leads to the intrusion of water molecules into the hydrophobic micropores. Once pressure is applied to the conventional ZIF-8 water system, intrusion leads to a substantial volume reduction, as shown in Figure 3a. The water intrusion process can substantially increase the compressibility of water by a factor of 20.^1^ Once the applied pressure is removed, the liquid returns to its initial volume, as the water molecules spontaneously diffuse from the ZIF-8 pores and back into the solution. This permits the compressible liquid to be reused and maintain the same compressibility over multiple cycles, as demonstrated in Figure 3a.

**Figure 3:**
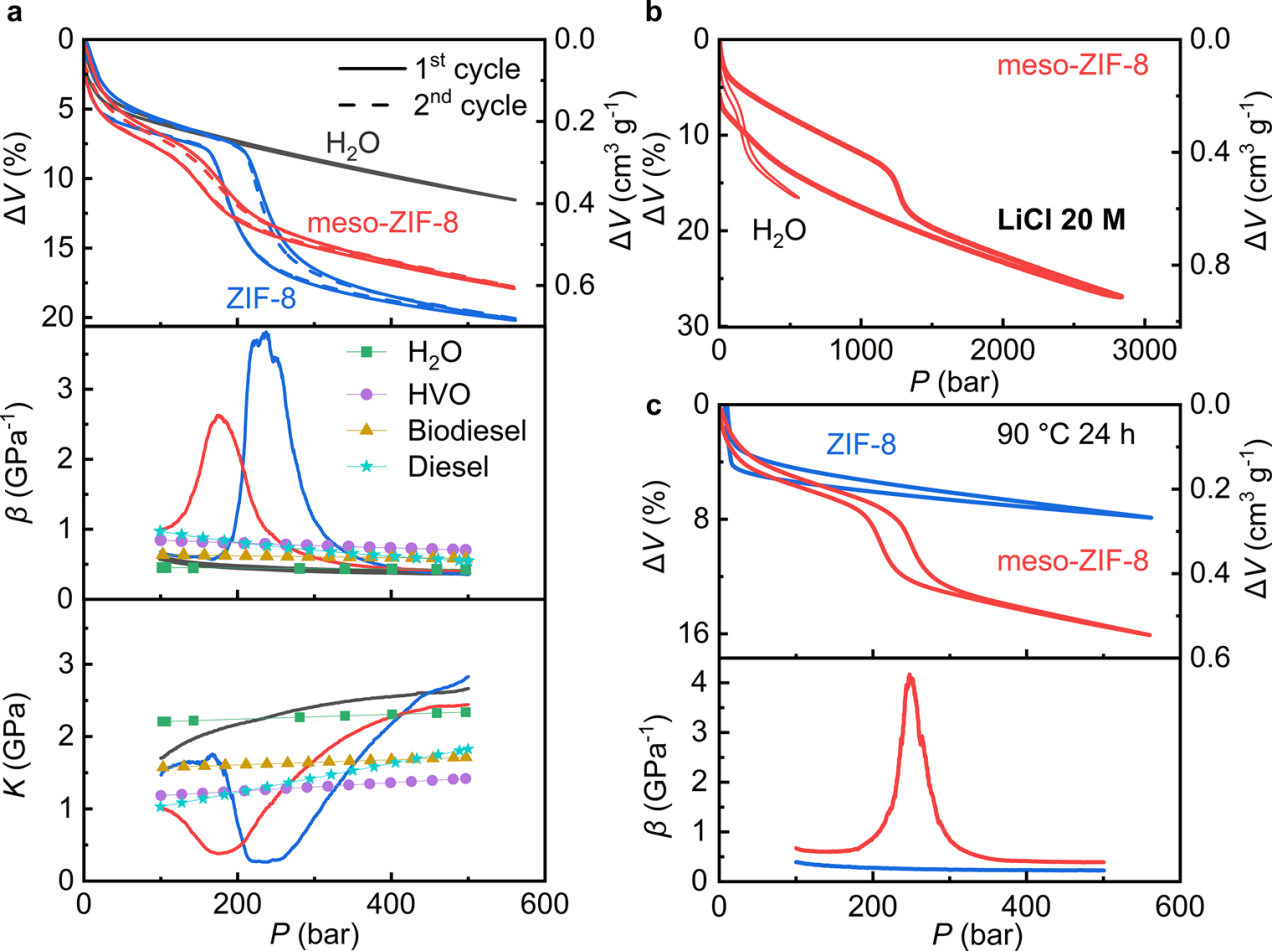
Compression tests on meso-ZIF-8 (red) and ZIF-8 (blue) in water at a concentration of 29.7 wt %. (a) Comparison between the two systems and conventional liquids such as water^100,101^ and three different oils,^67^ including their volume change in compression and decompression (with the solid line representing the first cycle and the dashed line representing the second cycle), and compressibility β (the relative volume change in response to the change in pressure) and bulk modulus *K* (reciprocal quantity of β) in compression at 100–500 bar. (b) Volume change of meso-ZIF-8 in water and 20 M LiCl solution under pressure. (c) Volume change and compressibility of the two systems under pressure after 24 h in 90 °C water. The pressure–volume change curves have been shifted on the vertical axis for the sake of clarity.

For meso-ZIF-8, upon applying pressure, if both the mesopores and micropores were being filled by water, two plateaus should be expected in the *P*–Δ*V* curves for the differently sized pores to be intruded at different pressures. However, only one plateau is observed in the *P*–Δ*V* curve (Figure 3a), indicating that water intrusion is only occurring in the hydrophobic micropores and that the hydrophilic mesopores have already been hydrated at the outset of the experiment before the pressure is applied. This is in line with a most recent publication on hierarchical ZIF-8,^55^ although for much bigger crystals from a different synthesis method. A further test was performed in a 20 M LiCl solution (Figure 3b), which has a much higher intrusion pressure owing to the strong interactions between water molecules in the solution and the concentrated ions.^21,65^ Still, only one plateau is observed, further indicating that the mesopores are very hydrophilic; they are already hydrated at ambient conditions, so only the micropores have water intrusion under an elevated pressure. Such high hydrophilicity revealed by the compression tests is in agreement with the material characterisations in the previous section (Figure 2a,b, Figure S3).

The meso-ZIF-8 water system shows a slightly smaller compressibility than the conventional ZIF-8 water system (Figure 3a), consistent with the reduced micropore volume in meso-ZIF-8 (Figure 1f) as well as a reported observation that ZIF-8 surface defects can be occupied by water at ambient pressure.^66^ The highest compressibility of the meso-ZIF-8 water system, 2.62 GPa^–1^ (corresponding to a bulk modulus of 0.38 GPa), is much greater than that of traditional liquids like pure water (0.45 GPa^–1^), hydrotreated vegetable oil (HVO, 0.81 GPa^–1^), biodiesel (0.62 GPa^–1^), and diesel (0.84 GPa^–1^) at the same applied pressures (Figure 3a). The working pressure of meso-ZIF-8 (175 bar), defined as the pressure at which the highest compressibility is obtained, is slightly lower than that of the ZIF-8 water system (237 bar), as shown in Figure 3a. The lower working pressure is likely due to the hydrophilic mesopores being hydrated, which permits more facile water intrusion into the nanopores. Previous studies have reported similar effects on small nanocrystals, which were also found to have a slightly lower intrusion pressure than larger crystals.^17,66^

The chemical stability of meso-ZIF-8 and conventional ZIF-8 was further examined by performing compression tests after 24 h at 90 °C and after 14 days at room temperature in water, respectively. Although both colloidal suspensions maintain their compressibility after 14 days at room temperature (Figure S5b), only the meso-ZIF-8 suspension maintains its compressibility after 24 h at 90 °C, while the conventional ZIF-8 suspension loses all compressibility (Figure 3c). This result agrees well with the stability tests reported in Figure 2e,f. It demonstrates that compressible liquids made of meso-ZIF-8 colloidal suspensions, despite the slightly reduced initial compressibility, are more stable and can be used for more extended periods than analogous suspensions containing conventional ZIF-8.

### 3.4 Kinetic Effect

Motivated by the potential application of the proposed compressible liquid in shock absorptions, we conducted compression tests at a higher compression rate (14 s^–1^ vs 3 × 10^–3^ s^–1^). We found a rate effect in both systems (Figure 4a): the working pressure and energy absorption increase by over 20% for both materials at the higher compression rate. According to our previous work,^17^ further growth can be expected under high-rate impacts at 10^3^ s^–1^. As the mesopores are likely hydrated at ambient conditions owing to their hydrophilic nature and the intrusion process involves only the hydrophobic micropores, these results did not show a significant influence of the mesopores on the rate effect. The water intrusion of the micropores is fully reversible (Figure 4a), indicating the potential to be a stable and reusable energy absorber.

**Figure 4:**
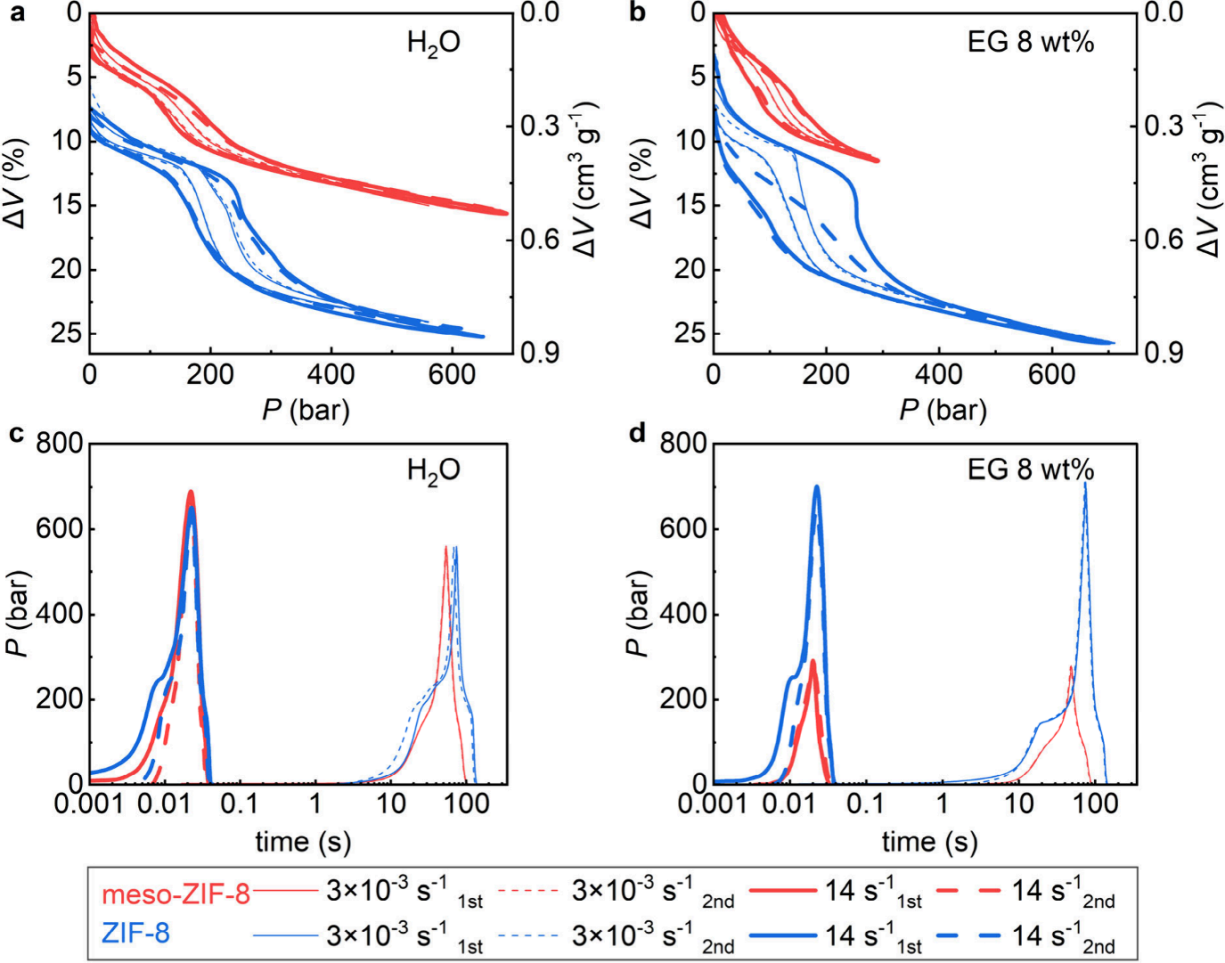
Kinetic tests on meso-ZIF-8 (red) and ZIF-8 (blue) in (a, c) water and (b, d) 8 wt % EG aqueous solution at a concentration of 29.7 wt %. (a) and (b) show the volume changes, and (c) and (d) show the pressure changes at two different strain rates for two cycles. The curves have been shifted along the volume change and time axes for the sake of clarity.

To investigate the influence of meso-porosity on dynamic compressibility further, larger molecules were introduced into the system to reduce the diffusion rate so that the influence of mesopores could be better established. Ethylene glycol (EG) was added to water to form an 8 wt % aqueous solution, substantially reducing the diffusion rate in conventional ZIF-8. As shown in Figure 4b, in compression tests performed at a lower compression rate, all the liquid diffused from the conventional ZIF-8 pores at the end of the first slow compression–decompression cycle, so the second cycle reproduces the first cycle’s behavior. In contrast, when a higher compression rate was employed, around half of the liquid remained within the material at the end of the faster cycle, as there was insufficient time for the liquid to diffuse out of conventional ZIF-8 at the interval between two pressure cycles. As shown in Figure 4c,d, a slow cycle takes 120 s, while a fast cycle takes only 40 ms. Suppose a sufficient gap is provided between cycles when employing the rapid rate (Figure S6). In that case, all of the liquid will diffuse out of the pores in conventional ZIF-8 even when a higher EG concentration is adopted.

In contrast, for meso-ZIF-8, almost all of the liquid diffuses out following both the slow and fast compression tests. The presence of mesopores increases the permeation rate of water molecules by increasing the surface area to volume ratio of the ZIF particles, allowing for more facile intrusion and extrusion of water molecules into the hydrophobic nanopores, reducing the average diffusion path length. The diffusion path length, i.e., the average distance a molecule must travel within a material before reaching its destination,^68^ in our case, is the distance for the liquid molecules to travel from the core of the crystal skeleton to its surfaces, which include the mesopore surface since it has been hydrated and remain filled with water during the process. With mesopores, more of the solid material is closer to the surface, allowing the liquid to escape faster. The enhanced mass transport permits a faster mechanical response in practical applications, which might be required to work against mechanical vibrations or repeated high-rate impacts.^17^ Previous studies in other areas have shown related phenomena, where additional meso-porosity enhances mass transfer in hierarchical MOFs,^44,69^ including water diffusion through hydrophilic membranes,^70^ although the process in this work is different due to the distinct surface properties between the micropores and the mesopores.

## 4 Conclusions

This study presents a highly stable compressible liquid, a uniform water suspension of ZIF nanocrystals containing hydrophobic micropores and hydrophilic mesopores. The hydrophobic micropores, kept empty at ambient conditions, offer a high compressibility due to the water intrusion process under pressure, much higher than the compressibility of conventional liquids. The hydrophilic mesopores, hydrated at ambient conditions, substantially improve the physical stability as a colloid and chemical stability in the crystals’ resistance to hydrolysis. This design strategy is demonstrated by stability and compressibility tests on a mesoporous ZIF-8 obtained by the soft template method, with a crystal size of ∼30 nm, a mesopore size of ∼3 nm, and an aperture size of ∼0.3 nm, and consistent results were obtained in reproducibility studies (Figure S7). Such compressible liquids can be used for a longer time, under harsher conditions, and undergo a larger number of compression cycles with the reversibility of water intrusion in the micropores. The introduction of hydrophilic mesopores slightly reduces the initial compressibility and water intrusion pressure but improves the kinetics of water transport that is desirable for highly dynamic cyclic loadings, such as in vibrations or repeated impacts. Future research can aim to better understand and control the mesoporous structures and surfaces and apply them to different MOFs, liquids, and environmental or loading conditions, leading to material design rules relevant to practical applications.

## Supplementary Material



## Data Availability

The experimental data sets generated during the current study are available from the online Figshare repository at https://doi.org/10.6084/m9.figshare.27369447.

## Notes

Compressibility measurement setup and additional experimental results including particle size and pore size distributions, ζ potential, FTIR, colloidal and chemical stability after 14 days, cyclic compressibility in ethylene glycol solution, and reproducibility studies (PDF)
