# Supplementary material for: Stable Compressible Liquids Made of Hierarchical MOF Nanocrystals
Source: ACS Appl Mater Interfaces. 2025 Apr 22;17(20):30161–9. doi: 10.1021/acsami.4c21181 (PMC12100603; doi:10.1021/acsami.4c21181)
Supplement: Supplementary file 1 [file am4c21181_si_001.pdf]

Supporting Information

Stable Compressible Liquids Made of Hierarchical MOF Nanocrystals

Heting Xiao [a,b], Xi-feng Liang [a], Wei Zhou [a], Hebin Jiang [b], Daniel S. Parsons [b], Haixia Yin [b], Bitao Lu [b], Yueting Sun [b*]

[a] School of Traffic & Transportation Engineering, Central South University, Changsha, Hunan, 410083, China

[b] School of Engineering, University of Birmingham, Edgbaston, Birmingham, West Midlands, B15 2TT, United Kingdom

*Email: y.sun.9@bham.ac.uk

Table of Contents

S1. Compressibility measurement set-up 3

S2. Particle size and pore size distributions 4

S3. ζ potential and FTIR results 6

S4. Colloidal stability after 14 days 7

S5. Chemical stability after 14 days in water at room temperature 8

S6. Cyclic compressibility of ZIF-8 with 15 wt% ethylene glycol (EG) solution. 9

S7. Reproducibility studies 10

References 12

S1. Compressibility measurement set-up

Figure S1 presents a schematic of the compressibility measurement set-up. The piston and pressure chamber are made from stainless steel, and the pistons have sealing rings to prevent leakage.

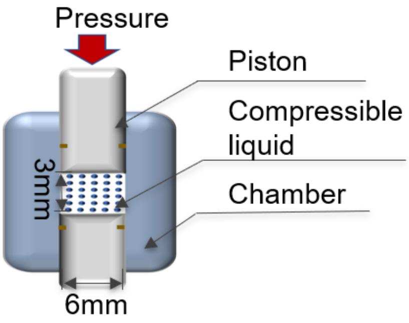

Figure S1. Compressibility measurement setup.

S2. Particle size and pore size distributions

The particle size and mesopore size were calculated using ImageJ software from the scanning electron microscopy (SEM) and transmission electron micrograph (TEM) presented in Figure S2. The size distribution histograms were derived through frequency statistical analysis and fitted with Gaussian distributions, while the cumulative frequency distributions of were fitted using Boltzmann functions.

For the particle size of meso-ZIF-8, the mean parameter μ of the Gaussian distribution is 32.1 nm with a standard deviation of 5.26 nm by counting 147 particles. The particle sizes at 10%, 50% and 90% of its cumulative frequency are 25 nm, 31.2 nm and 37.8 nm, respectively (Figure S2a, c).

For the particle size of conventional ZIF-8, the mean parameter μ of the Gaussian distribution is 32.9 nm with a standard deviation of 4.50 nm by counting 130 particles. The particle sizes at 10%, 50% and 90% of its cumulative frequency are 26.9 nm, 32.2 nm and 37.8 nm, respectively (Figure S2b, d). Therefore, the particle size of meso-ZIF-8 is very close to that of conventional ZIF-8.

For the mesopore size of meso-ZIF-8, the mean parameter μ of the Gaussian distribution is 2.3 nm with a standard deviation of 0.74 nm by counting 119 pores (Figure S2e-f). The diameter at 90% of its cumulative frequency is 3.3 nm (Figure S2e).

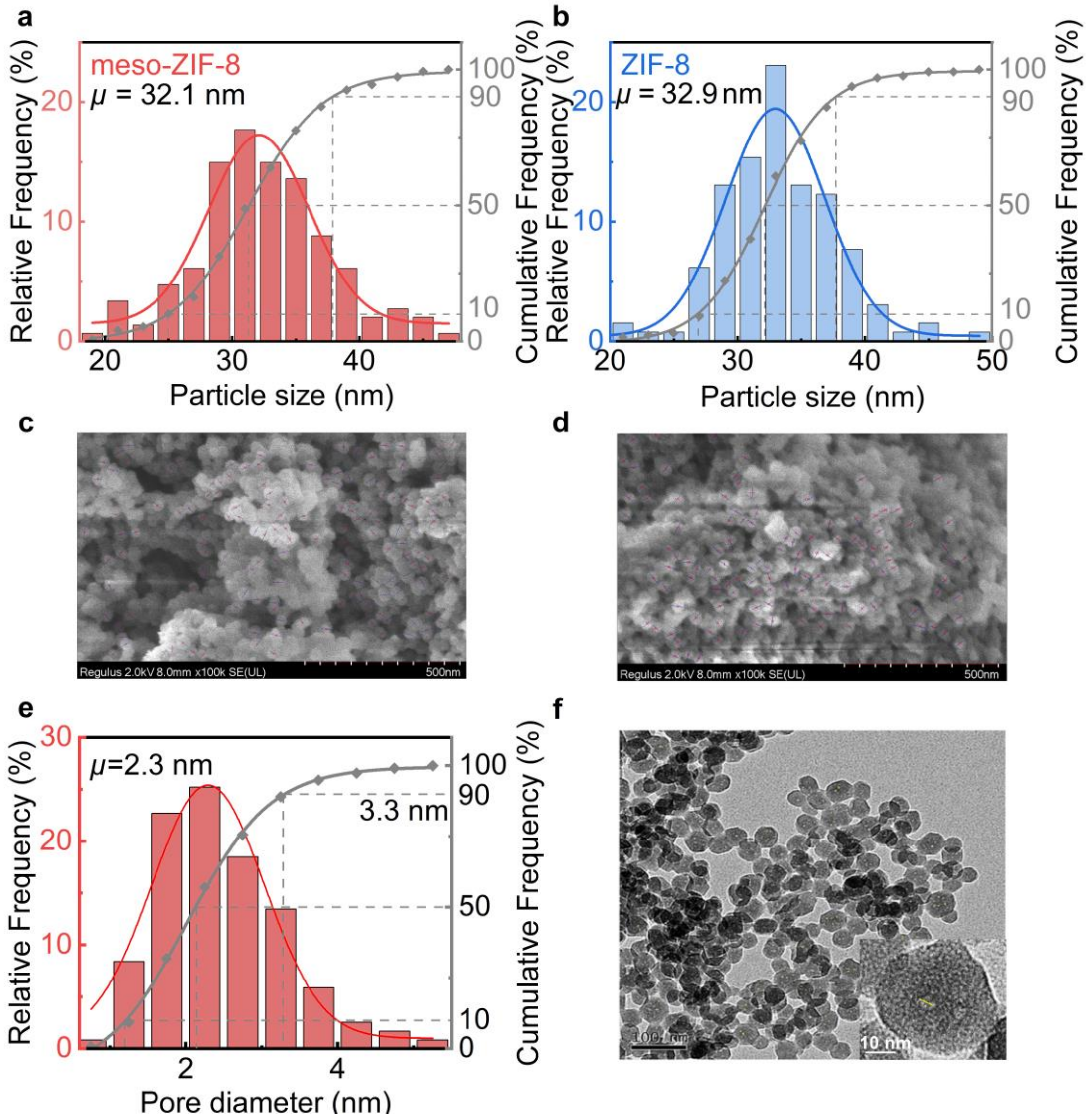

Figure S2. Particle size and pore diameter distribution of meso-ZIF-8 and conventional ZIF-8, calculated from SEM and TEM images using ImageJ. (a) Particle size distribution of meso-ZIF-8. (b) Particle size distribution of conventional ZIF-8. (c) SEM of meso-ZIF-8. (d) SEM of conventional ZIF-8. (e) Mesopore size distribution of meso-ZIF-8. (f) TEM of meso-ZIF-8.

S3. ζ potential and FTIR results

The ζ potential results in Figure S3a show that the average surface charge of ZIF-8 is 19.7 mV, consistent with published results.[1-3] The average surface charge of meso-ZIF-8 is -6.3 mV, probably due to the zinc-rich surface capping that occurs upon removing CTAB and L-histidine templates when zinc ions bind to hydroxyl groups in water. This hypothesis is supported by the reported ζ potential of zinc hydroxide -10 mV at pH 7,[4] and the O-H peak on the FTIR of the meso-ZIF-8 sample (Figure S3b).[5] These hydroxyl groups make meso-ZIF-8 more hydrophilic. Besides, it has been reported that 3D porosity and pore diameter can affect ζ potential,[6] so the presence of mesopores and reduced surface area may also have contributed to the decreased surface charge density of the meso-ZIF-8 sample.

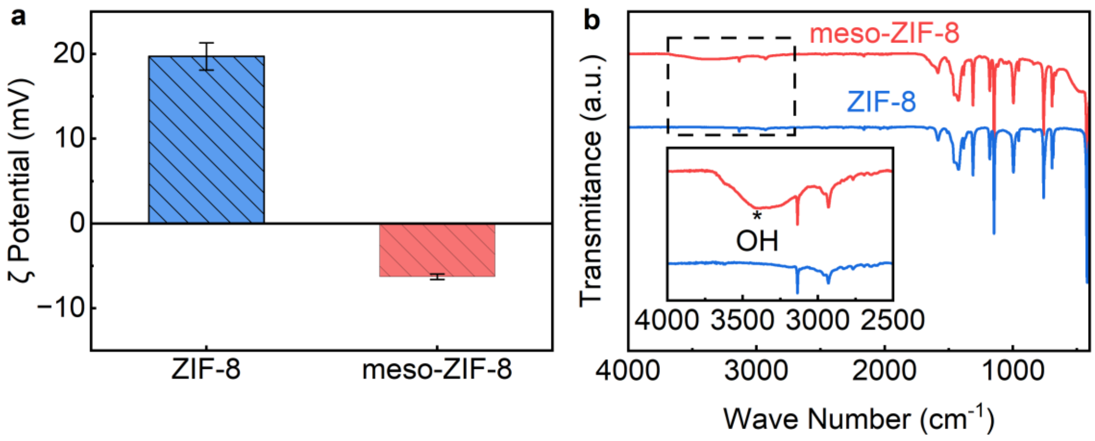

Figure S3. ζ potential and FTIR result of meso-ZIF-8 and ZIF-8. (a) ζ potential in water. (b) FTIR results.

S4. Colloidal stability after 14 days

After 14 days, the meso-ZIF-8 water suspension will start losing its uniformity, as recorded in Figure S4 on day 15. However, this is still much more stable than the conventional ZIF-8 suspension, which undergoes a complete separation within just 1 day (Figure 2d in the manuscript). As described in the manuscript, the colloidal stabilities of the system can be increased further by various methods,[7, 8] but since achieving ‘permanent’ colloidal stability is not our goal, further optimisation is outside the scope of this study.

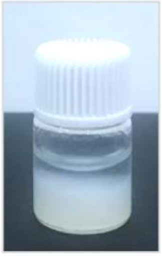

Figure S4. Colloidal stability of meso-ZIF-8 on day 15 with the concentration of 1.6 wt% aqueous solution at room temperature. This result used a new batch of sample (batch 2 in Figure S7) tested in a slightly smaller sample vial compared to Figure 2d of the manuscript, but followed the same synthesis and testing process.

S5. Chemical stability after 14 days in water at room temperature

The stability of ZIF-8 and meso-ZIF-8 in aqueous media has also been evaluated by adding the ZIF (1.6 wt%) to deionised water for 14 days at ambient temperature. PXRD patterns were recorded on the recovered solids (Figure S3a), and compressibility measurements were conducted on the suspensions (Figure S3b). PXRD shows that ZIF-8 and meso-ZIF-8 maintain their main structure after immersion in aqueous media. However, ZIF-8 shows a significant additional reflection at $2\theta \approx 12°$ (asterisk in Figure S3), indicating the formation of an extra phase. The compressibility measurements do not show significant changes after 14 days in either of these two samples.

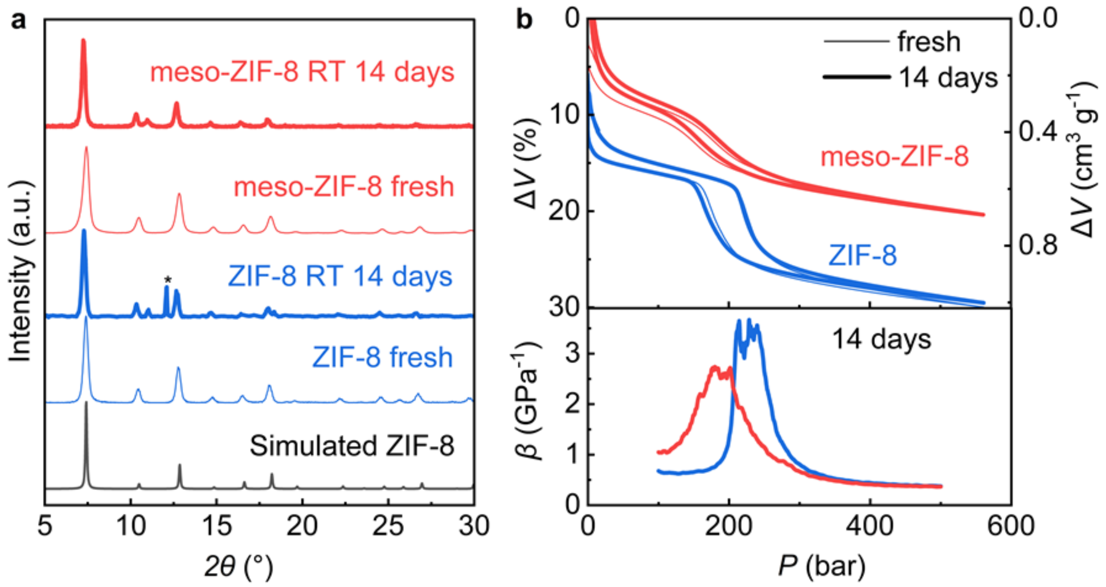

Figure S5. Stability and compressibility tests of meso-ZIF-8 (red) and ZIF-8 (blue) after 14 days in liquid water at room temperature. (a) PXRD results (collected after being in water at a concentration of 1.6 wt%). (b) Volume change and compressibility under pressure (in water at concentration of 29.7 wt%). The pressure - volume change curves have been shifted on the vertical axis for clarity.

S6. Cyclic compressibility of ZIF-8 with 15 wt% ethylene glycol (EG) solution

Compression tests were performed on conventional ZIF-8 with a 15 wt% EG aqueous solution at a strain rate of 14 s^{-1} for four cycles, with a 5 min gap between the 2nd and 3rd cycles. Figure S6 shows that this 5 min allows the liquid to extrude from the ZIF-8 pores, and therefore, the ZIF-8 porosity and liquid compressibility are recovered in the 3rd cycle. This contrasts with the substantially reduced compressibility in the 2nd and 4th cycles, which immediately follow the 1st and 3rd cycles, respectively.

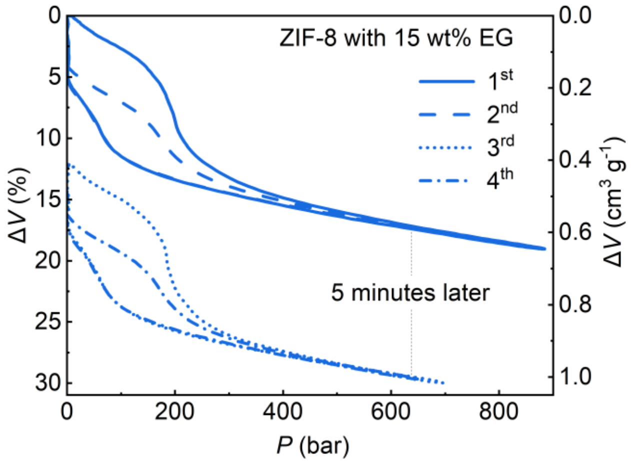

Figure S6. Compression tests on conventional ZIF-8 with a 15 wt% EG aqueous solution at a strain rate of 14 s^{-1}, starting with two consecutive cycles, followed by 5 min of relaxation, and another two consecutive cycles. The curves have been shifted vertically for the sake of clarity.

S7. Reproducibility studies

We synthesised two batches of meso-ZIF-8 and ZIF-8 and carried out experiments in line with Figures 1-3 of the manuscript to prove that the reported methods can be reproduced. Firstly, we performed PXRD (Figure S7a) and FTIR (Figure S7b) on the synthesised batches, demonstrating reproducible synthesis processes. We then tested their compressibility with water (Figure S7c-d) and gained consistent results between the two batches with minimal discrepancy. Furthermore, a physical stability test was conducted on the batch 2 sample (Figure S7e), showing a stable dispersion of meso-ZIF-8 in water, consistent with the observation on the batch 1 sample presented in Figure 2d of the manuscript. PXRD patterns after 24 h in water at 90 °C (Figure S7f) also demonstrate the consistent chemical stability between the two batches of materials. By repeating these experiments, we are confident that the approach and results presented in this work can be reproduced by others interested in further studies in this direction.

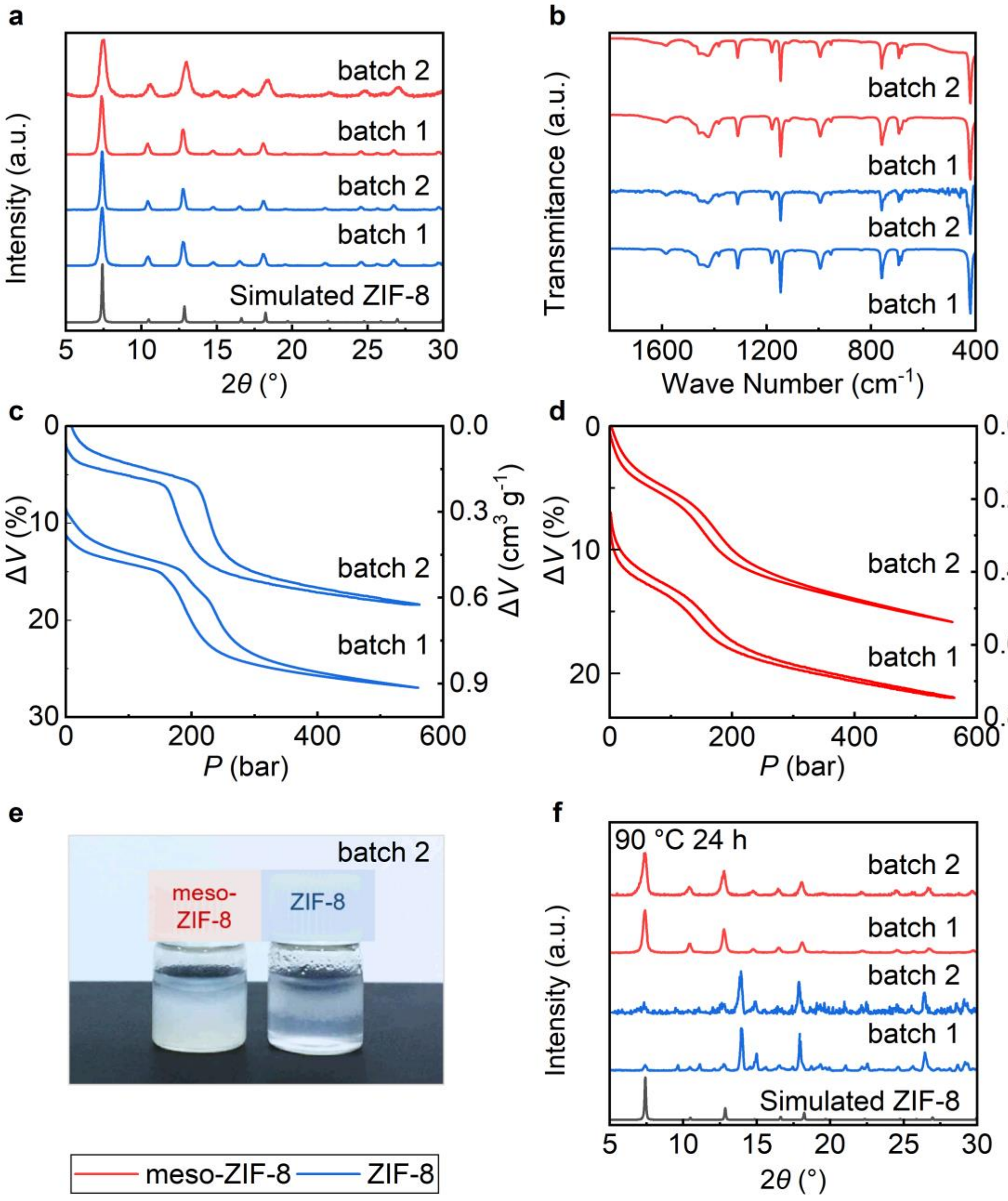

Figure S7. Reproducibility tests on ZIF-8 and meso-ZIF-8 by comparing two different batches of samples, including (a) PXRD, (b) FTIR, (c-d) compressibility tests, (e) photos of the batch 2 sample after 1 day in water at a concentration of 1.6 wt%, and (f) PXRD after 24 h in water at 90 °C at a concentration of 29.7 wt%.

References

(1) Jian, M.; Liu, B.; Zhang, G.; Liu, R.; Zhang, X. Adsorptive removal of arsenic from aqueous solution by zeolitic imidazolate framework-8 (ZIF-8) nanoparticles. *Colloids and Surfaces A: Physicochemical and Engineering Aspects* **2015**, *465*, 67-76.

(2) Jongert, T. K.; Slowinski, I. A.; Dao, B.; Cortez, V. H.; Gredig, T.; Plascencia, N. D.; Tian, F. Zeta Potential and Size Analysis of Zeolitic Imidazolate Framework-8 Nanocrystals Prepared by Surfactant-Assisted Synthesis. *Langmuir* **2024**, *40* (12), 6138-6148.

(3) Jia, Z.; Wu, G.; Wu, D.; Tong, Z.; Winston Ho, W. Preparation of ultra-stable ZIF-8 dispersions in water and ethanol. *Journal of Porous Materials* **2017**, *24*, 1655-1660.

(4) Solís–Rodríguez, R.; Pérez–Garibay, R.; Alonso–González, O.; Mendieta–George, D. Enhancing the arsenic adsorption by controlling the zeta potential of Zn (OH) 2 flocs. *Journal of Environmental Chemical Engineering* **2021**, *9* (5), 106300.

(5) Khan, S. R.; Abid, S.; Jamil, S.; Aqib, A. I.; Faisal, M. N.; Ashraf Janjua, M. R. S. Layer by layer assembly of zinc oxide nanotubes and nanoflowers as catalyst for separate and simultaneous catalytic degradation of dyes and fuel additive. *ChemistrySelect* **2019**, *4* (19), 5548-5559.

(6) Yakin, F. E.; Barisik, M.; Sen, T. Pore size and porosity dependent zeta potentials of mesoporous silica nanoparticles. *The Journal of Physical Chemistry C* **2020**, *124* (36), 19579-19587.

(7) Zatz, J. Physical stability of suspensions. *Journal of the Society of Cosmetic Chemists* **1985**, *36* (6), 393-411.

(8) Cahir, J.; Tsang, M. Y.; Lai, B.; Hughes, D.; Alam, M. A.; Jacquemin, J.; Rooney, D.; James, S. L. Type 3 porous liquids based on non-ionic liquid phases–a broad and tailorable platform of selective, fluid gas sorbents. *Chemical Science* **2020**, *11* (8), 2077-2084.
